# Latest clinical frontiers related to autism diagnostic strategies

**DOI:** 10.1016/j.xcrm.2024.101916

**Published:** 2025-01-28

**Authors:** Samuele Cortese, Alessio Bellato, Alessandra Gabellone, Lucia Marzulli, Emilia Matera, Valeria Parlatini, Maria Giuseppina Petruzzelli, Antonio M. Persico, Richard Delorme, Paolo Fusar-Poli, Corentin J. Gosling, Marco Solmi, Lucia Margari

**Affiliations:** 1 Developmental EPI (Evidence synthesis, Prediction, Implementation) Lab, Centre for Innovation in Mental Health, School of Psychology, Faculty of Environmental and Life Sciences, University of Southampton, Southampton, UK; 2 Clinical and Experimental Sciences (CNS and Psychiatry), Faculty of Medicine, University of Southampton, Southampton, UK; 3 Hampshire and Isle of Wight NHS Foundation Trust, Southampton, UK; 4 Hassenfeld Children’s Hospital at NYU Langone, New York University Child Study Center, New York City, NY, USA; 5 DiMePRe-J-Department of Precision and Rigenerative Medicine-Jonic Area, University of Bari "Aldo Moro", Bari, Italy; 6 Institute for Life Sciences, University of Southampton, Southampton, UK; 7 Mind and Neurodevelopment (MiND) Interdisciplinary Cluster, University of Nottingham, Malaysia, University of Nottingham Malaysia, Semenyih, Malaysia; 8 DIBRAIN - Department of Biomedicine Translational and Neuroscience, University of Bari “Aldo Moro”, Bari, Italy; 9 Department of Biomedical, Metabolic and Neural Sciences, University of Modena and Reggio Emilia, & Child & Adolescent Neuropsychiatry Program, Modena University Hospital, Modena, Italy; 10 Child and Adolescent Psychiatry Department & Child Brain Institute, Robert Debré Hospital, Paris Cité University, Paris, France; 11 Early Psychosis: Interventions and Clinical-detection (EPIC) Lab, Department of Psychosis Studies, King’s College London, London, UK; 12 Department of Brain and Behavioral Sciences, University of Pavia, Pavia, Italy; 13 Outreach and Support in South-London (OASIS) Service, South London and Maudlsey (SLaM) NHS Foundation Trust, London, UK; 14 Department of Psychiatry and Psychotherapy, University Hospital, Ludwig-Maximilian-University (LMU), Munich, Germany; 15 Université Paris Nanterre, Laboratoire DysCo, Nanterre, France; 16 Université de Paris Cite', Laboratoire de Psychopathologie et Processus de Santé, Boulogne-Billancourt, France; 17 SCIENCES Lab, Department of Psychiatry, University of Ottawa, Ottawa, ON, Canada; 18 Regional Centre for the Treatment of Eating Disorders and On Track: The Champlain First Episode Psychosis Program, Department of Mental Health, The Ottawa Hospital, Ottawa, ON, Canada; 19 Ottawa Hospital Research Institute (OHRI) Clinical Epidemiology Program University of Ottawa, Ottawa, ON, Canada; 20 Department of Child and Adolescent Psychiatry, Charité Universitätsmedizin, Berlin, Germany

**Keywords:** autism, diagnosis, biomarkers, machine learning, artificial intelligence, genetics, telemedicine

## Abstract

The diagnosis of autism is currently based on the developmental history, direct observation of behavior, and reported symptoms, supplemented by rating scales/interviews/structured observational evaluations—which is influenced by the clinician’s knowledge and experience—with no established diagnostic biomarkers. A growing body of research has been conducted over the past decades to improve diagnostic accuracy. Here, we provide an overview of the current diagnostic assessment process as well as of recent and ongoing developments to support diagnosis in terms of genetic evaluation, telemedicine, digital technologies, use of machine learning/artificial intelligence, and research on candidate diagnostic biomarkers. Genetic testing can meaningfully contribute to the assessment process, but caution is required when interpreting negative results, and more work is needed to strengthen the transferability of genetic information into clinical practice. Digital diagnostic and machine-learning-based analyses are emerging as promising approaches, but larger and more robust studies are needed. To date, there are no available diagnostic biomarkers. Moving forward, international collaborations may help develop multimodal datasets to identify biomarkers, ensure reproducibility, and support clinical translation.

## Graphical abstract

**Figure:**
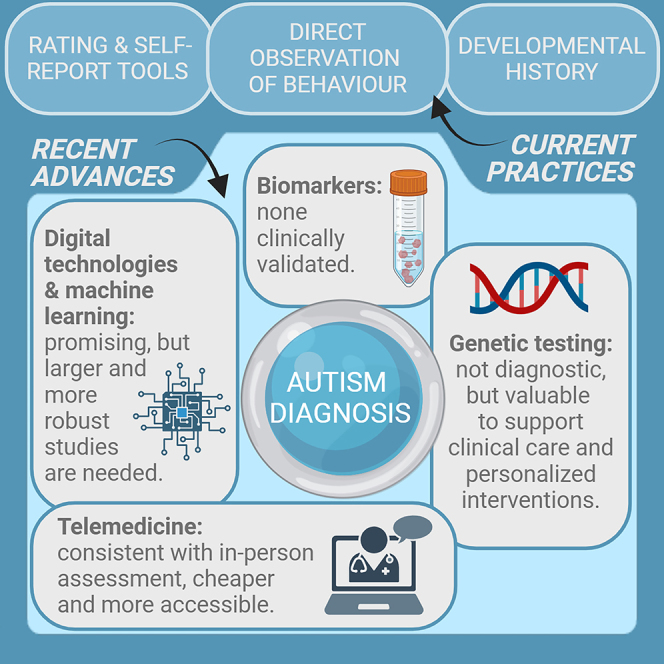

## Teaser

Cortese et al. reviewed current practices and research developments to support the diagnostic process of autism. Overall, genetic testing is not yet diagnostic but provides valuable background information to support clinical care and personalized interventions, while telemedicine, digital diagnostics, and machine-learning-based approaches appear promising but warrant further investigation via larger and more rigorous studies aimed to identify viable biomarkers.

## Introduction: Definition and conceptualization of autism

Autism, characterized by alterations in social interaction/communication and repetitive behaviors/interests,^1^ is one of the most common neurodevelopmental conditions.^2^ Although meta-analytic evidence based on a limited number of studies indicates that the peak age of onset might occur around the early years of life,^3^ autism begins much earlier, potentially during prenatal development.^4^^,^^5^ The average age of first diagnosis varies across countries, with the most recent estimates of median age at the earliest known diagnosis being 49 months in the United States.^6^ An autism diagnosis made in childhood persists in adulthood in a sizable portion of cases.^7^

The conceptualization of autism has been constantly evolving, moving from a narrow initial categorization among the childhood psychoses to its current, broader definition as a “spectrum”—i.e., autism spectrum disorder (ASD).^8^^,^^9^ This evolution reflects efforts to enhance the reliability of the diagnosis while preserving its validity. However, broadening the construct of autism raises issues around its boundaries with other neurodevelopmental conditions and typical development. There have also been concerns that broadening the construct of autism may inflate the diagnostic rate and hinder the understanding of its causes and developmental pathways.^10^

The latest versions of the two most frequently used classification systems in mental health, namely the Diagnostic and Statistical Manual of Mental Disorders (DSM)-5-Text Revision (TR)^11^ and the International Classification of Diseases and Related Health Problems (ICD)-11,^12^ classify ASD within the broader category of “neurodevelopmental disorders,” with onset of symptoms usually during the early years of life (Table S1). Both classification systems require persistent alterations in two core domains, namely (1) social communication/social interactions (e.g., struggling to engage in reciprocal conversation) and (2) restricted, stereotyped, and repetitive patterns of behavior/interests/activities (e.g., pervasive interest in calendars/dates).

While the current classification systems refer to ASD as a categorical diagnosis, it has been highlighted that the symptoms of autism lie on the extreme of a continuous distribution of traits (the *dimensional* view). The current conceptualization of autism leads to a substantial range of clinical variability and impairment in everyday life functioning, which highlights the need for diagnostic approaches that capture specific clinical features of each individual, to inform personalized management strategies. Recent classifications stress that ASD behaviors/symptoms can range from overtly manifest to more subtle, thus only becoming evident when demands of the context exceed the capacity of the individual. Notably, even though the symptoms of ASD are expected to emerge typically in early childhood,^13^ they may not become fully manifest until later in life, when social demands exceed an individual’s capacities.^11^ Therefore, in some cases, the diagnosis is made for the first time beyond childhood. As such, it is essential to appreciate that the clinical diagnosis of ASD is only appropriate when there are significant impairments associated with the symptoms, and/or when the individual makes significant efforts to minimize the impairment associated with the symptoms and meet expected functioning levels.

Subtle yet important differences exist between the DSM-5-TR and the ICD-11 criteria in their conceptualization of ASD (Table S1). The DSM-5-TR diagnostic criteria are more oriented toward a medical model of brain illness, specifying the number of required observable behavioral symptoms needed to identify the core symptoms and providing descriptions of severity levels. The ICD-11 moved toward a social model of disability, giving more emphasis to the inner experience of “diversity” and to the poor fit between individual’s characteristics and demands by the environment.^8^ This reflects the ongoing tendency to move beyond a medical conceptualization of autism, which sees disabilities as inherent to the individual, toward a social perspective view (i.e., the disability is caused by barriers imposed to the person by society). This has been prompted by the *neurodiversity movement*,*^14^* a social justice and self-representative movement stemming from the disability rights, which challenges a narrow medical conceptualization of autism, considering it as the expression of human diversity. In our view, rather than viewing the medical and social models of autism as mutually exclusive, blending them and acknowledging both differences and disability may be a promising way forward.

There is a substantial variability in the administrative prevalence of ASD (i.e., the one that is determined based on administrative records such as billing records, or other documents that include an ICD code) across geographic regions. The global age-standardized prevalence of ASD across countries has been reported at 0.37% in the most recent estimate from the Global Burden of Disease.^15^ However, for instance, about 1 in 36 children with autism were identified in the USA in 2020 as reported by the Centers for Disease Control and Prevention.^16^ This variability is likely accounted for by a plethora of factors, including the lack of an objective diagnostic test, socio-cultural factors related to variations in cultural acceptance of mental health conditions, diseases and disorders, variations in digital methods that allow for rapid and accurate ascertainment of clinical and service records that document ASD diagnosis (i.e., recordkeeping and digital tracking of diagnoses in medical health systems in some countries is not consistently available, making accurate tracking challenging), differences in medical training and awareness of autism among clinical professionals, and differences in economic resources required to diagnose and treat autism. Despite the complexity of these factors, improving the diagnostic accuracy itself is key, to increase the chances that individuals with ASD get the right support. To this end, a growing body of research has been conducted over the past decades.

Here, we provide an overview of the latest clinical frontiers related to autism diagnostic strategies, focusing on the current clinical diagnostic assessment process across the lifespan as well as on recent and ongoing developments in terms of genetic evaluation, telemedicine, digital technologies, use of machine learning (ML)/artificial intelligence (AI), and research on candidate diagnostic biomarkers. A review of the literature on current diagnostic models (e.g., traditional center-based multidisciplinary assessment vs. single-discipline mentored community assessments) is beyond the scope of the present article.

Of note, here we use the term “ASD” in line with the formal current terminology in classification systems and with the majority of published scientific studies. However, currently, other terms, such as *autism spectrum condition* or simply *autism*, which reflect the influence of the neurodiversity movement, are also used. By no means does our use of ASD imply that we disregard the needs expressed by this movement.

The present review was not intended as a systematic review with a pre-specified protocol, including a search strategy and study quality assessment, but rather as a narrative review. Nonetheless, to ensure we did not miss any key studies in the field, we conducted multiple searches (up to March 28^th^, 2024) in PubMed using a combination of terms related to *autism* (or equivalent terms such as *autism spectrum disorder*, *Asperger’s*, *pervasive developmental disorders*), *diagnosis* (or related terms such as *assessment*), and specific terms related to *genetics*, *telemedicine*, *digital technologies*, *artificial intelligence/machine learning*, and *biomarkers*.

## Assessment of ASD: Current approaches

The assessment and diagnostic process of ASD can be a complex and challenging clinical task. While a multidisciplinary team approach is recommended, recent guidance suggests that, in order not to delay access to interventions, a trained healthcare provider comfortable with the assessment of autism clinical criteria can make an initial autism diagnosis, particularly when the diagnosis appears uncomplicated.^17^^,^^18^

According to the current conceptualization, the specific aim of the diagnostic process for ASD is to define whether an individual meets the behavioral diagnostic criteria for a formal diagnosis, within the context of a broader neurodevelopmental, behavioral, medical, and psychosocial assessment.^19^ To achieve this purpose, information is gathered through (1) a detailed developmental, medical, and psychosocial history, typically obtained from parents/carers; (2) direct observation of behavior, including social interactions, communications, and repetitive/stereotyped behaviors in different settings with familiar and unfamiliar individuals; and (3) subjective description—especially for adolescents and adults—of one’s inner perception of social functioning and interests.^1^ A clinical diagnosis of autism could be made by 18–24 months, with early features such as atypicality in (joint) attention, prelinguistic communication, social engagement, and sensorimotor processing observable in infancy.^20^ However, diagnostic instability has been observed in early life more than at any other age. For instance, in one study, a diagnosis of ASD established at 36 months of age was missed at 18 months in 63% of cases, while children diagnosed at the age of 18 presented a stability of diagnosis at 36 months of 93%^21^—even though, as that study sample referred to a group of younger siblings who were followed regardless of clinical concerns/referral, the study results may not be representative of the general population with ASD. Notably, a cohort study of 1,269 toddlers reported an overall stability of 0.84 for the autism diagnosis formulated between 12 and 36 months of age, higher than in other clinical groups.^22^

While several guidelines (e.g., those from the National Institute for Health and Care Excellence^19^) recommend routine systematic monitoring of early development of all children (“developmental surveillance”), the American Academy of Pediatrics currently recommends standardized universal autism screening (in addition to developmental surveillance) at 18 and 24 months using the parent-reported Modified Checklist for Autism in Toddlers. This tool has adequate meta-analytically pooled sensitivity (0.83, 95% confidence interval [CI] 0.77–0.88) and specificity (0.94, 0.89–0.97),^23^ particularly in children aged 18–30 months.

Beyond the screening of autistic symptoms at early age, the formal diagnostic process based on a combination of structured and semi-structured tools can improve diagnostic accuracy for ASD.^24^ These tools range from checklist/questionnaires for screening and rapid ascertainment of symptom severity, such as the *Social Communication Questionnaire*, to structured diagnostic interviews, including the *Autism Diagnostic Interview*, *Revised* (ADI-R), the *Developmental*, *Dimensional and Diagnostic Interview*, the *Childhood Autism Rating Scale*, *second edition* (CARS-2), and observational evaluation tools such as *the Autism Diagnostic Observation Schedule*, *second edition* (ADOS-2) (Table 1). Meta-analytic evidence showed that the sensitivity and specificity, respectively, of these tools for the diagnosis of ASD in preschoolers were as follows: ADOS-2: 0.94 (95% CI: 0.89–0.97) and 0.80 (0.68–0.88), CARS: 0.80 (0.61–0.91) and 0.88 (0.64–0.96), and ADI-R: 0.52 (0.32–0.71) and 0.84 (0.61–0.95).^25^ Overall, the performance of the ADOS-2 was superior to that of the ADI-R in children and adolescents (<18 years), although only few studies provided a direct comparison of the diagnostic accuracy of these instruments. For the ADOS-2, sensitivity and specificity ranged from 0.89 to 0.92 and 0.81 to 0.85, respectively. Studies comparing the accuracy of the ADOS-2 in research and clinical settings reported mixed evidence. Sensitivity and specificity of the ADI-R were 0.75 and 0.82, respectively, with higher specificity in research samples (research = 0.85, clinical = 0.72), although sparse clinical studies have been conducted to date.^26^ These findings indicate that relying solely on these tools for the diagnosis can lead to false positives and negatives. Additional evidence indicates that diagnoses made with standardized evaluation are more reliable across sites and more valid over time than single-clinician assessments.^27^ However, the use of ASD-specific diagnostic tools is often expensive and time-consuming for mental health services and requires a formal training of interviewers. Furthermore, even when administered by specifically trained staff, the various tools have a limited ability to correctly identify individuals whose diagnosis is more uncertain.^28^ Crucially, it should be pointed out that these tools were initially devised to help clinicians gather corroborative information, not to replace clinical judgment or serve as a triage system to determine access to services. Indeed, scores on these tools are highly dependent on how the tools are administered and interpreted, and hence their administration requires clinical expertise.^29^

**Table 1:** Examples of standardized instruments for the assessment of autism spectrum disorder

Standardized assessment instruments	
Estimate level of verbal and non-verbal development • Apply at least one verbal and one non-verbal problem-solving test from a cognitive or developmental assessment	brief screening: WASI, SB5 Routing subtests, KBIT, BINS, INTER-NDA more specific screening or comprehensive assessment: WPPSI, WISC, WAIS, DAS, RPM, MSEL, Bayley, M-P-R, PEP, RNDA
Estimate level of language functioning • Observe and ask caregivers about complexity of speech (e.g., few to no words, some words up to simple phrases, flexible phrases, or fluent)	brief screening**:** CELF screening test, PLS screening, CDI more specific screening or comprehensive assessment: CELF, PLS, OSEL
Assess ASD signs by history and in current daily life • Gather information from parents or other caregivers • If possible, gather information from multiple settings (e.g., home and school)	brief screening: SRS, SCQ, M-CHAT, AQ, CCC, PAAS, CAST, ASRS, ASSQ, SCDC more specific screening or comprehensive assessment: ADI-R, DISCO, 3-di
Assess ASD signs by observational assessment • Directly observe and interact with the individual in structured and unstructured interactive activities appropriate to developmental level	brief screening: STAT, SORF, AOSI, CARS, BOSCC, AMSE, TIDOS more specific screening or comprehensive assessment: ADOS
Estimate level of adaptive functioning • Ask questions about the individual’s adaptive functioning at home and in other everyday life settings	brief screening: SDQ impact supplement, WHODAS more specific screening or comprehensive assessment: VABS, ABAS

3-di, Developmental, Dimensional and Diagnostic Interview; ABAS, Adaptive Behavior Assessment System; ADI-R, Autism Diagnostic Interview, Revised; ADOS, Autism Diagnostic Observation Schedule; AMSE, Autism Mental Status Exam; AOSI, Autism Observation Scale for Infants; AQ, Autism-Spectrum Quotient; ASRS, Autism Spectrum Rating Scales; ASSQ, Autism Spectrum Screening Questionnaire; Bayley, Bayley Scales of Infant and Toddler Development; BINS, Bayley Infant Neurodevelopment Screener; BOSCC, Brief Observation of Social Communication Change; CARS, Childhood Autism Rating Scale; CAST, Childhood Autism Spectrum Test; CCC, Children’s Communication Checklist; CDI, MacArthur-Bates Communicative Development Inventories; CELF, Clinical Evaluation of Language Fundamentals; DAS, Differential Ability Scales; DISCO, Diagnostic Interview for Social and Communication Disorders; INTER-NDA, INTERGROWTH-21^st^ Neurodevelopment Assessment; KBIT, Kaufman Brief Intelligence Test; M-CHAT, Modified Checklist for Autism in Toddlers; M-P-R, Merrill-Palmer-Revised scales; MSEL, Mullen Scales of Early Learning; OSEL, Observation of Spontaneous Expressive Language; PAAS, pictorial autism assessment schedule; PEP, Psychoeducational Profile; PLS, Preschool Language Scales; RNDA, Rapid Neurodevelopmental Assessment; RPM, Raven’s Progressive Matrices; SB5, Stanford-Binet Intelligence Scale, fifth edition; SCDC, Social and Communication Disorders Checklist; SCQ, Social Communication Questionnaire; SDQ, Strengths and Difficulties Questionnaire; SORF, Systematic Observation of Red Flags; SRS, Social Responsiveness Scale; STAT, Screening Tool for Autism in Toddlers & Young Children; TIDOS, Three-item Direct Observation Screen; VABS, Vineland Adaptive Behavior Scales; WAIS, Wechsler Adult Intelligence Scale; WASI, Wechsler Abbreviated Scale of Intelligence; WHODAS, WHO Disability Assessment Schedule; WISC, Wechsler Intelligence Scale for Children; WPPSI, Wechsler Preschool and Primary Scale of Intelligence. Modified from Lord et al.^30^

An important challenge in the diagnostic process is delineating the diagnostic boundaries of ASD. Since autistic traits and/or features are continuously distributed in the general population, a fundamental but contentious issue is how the clinical thresholds are established, alongside functional impairment, for the purpose of a formal diagnosis of ASD.^31^

Another important aspect in the assessment of ASD relates to its interplay with additional neurodevelopmental conditions, impacting more globally on developmental trajectories. The differential diagnosis with global developmental delay and intellectual disability is particularly relevant, both for their high frequency and because they require that autistic features be “weighed” relative to the overall developmental/functional profile. According to the DSM-5(TR), the presence of global developmental delay or intellectual disability excludes a formal diagnosis of ASD, unless “social communication is below that expected for general developmental level.” Hence, if all functional domains are equally delayed or affected, it is unjustified to specifically underscore deficits in social communication over other deficits by giving an ASD diagnosis. However, if social interaction and communication/language development-related dimensions are more profoundly affected, compared to motor development and overall performance, then an ASD diagnosis may be justified, accompanied by specifiers regarding intellectual and/or language impairment. In this case, for the diagnosis, it is especially valuable to perform a psychometric assessment (using a scale such as the Mullen,^32^ the Griffiths,^33^ or the Bailey^34^) of the developmental abilities among the functional domains assessed by the scale and compare the intra-domain homogeneity.

A topic of increasing interest concerns gender differences in the clinical presentation of autistic individuals. Growing evidence suggests gender-dependent and gender-specific mechanisms contributing to differential phenotypes in ASD, with a consistent presence of male bias.^35^ Explanations include the male-reference conceptualization of ASD as well as the growing evidence of “camouflaging” behavior in females, masking their autistic traits by overcompensating in other areas,^36^^,^^37^^,^^38^ at the expense of requiring a major psychological effort, enhancing the risk of developing depression in adolescence or adulthood.

The evaluation process should also consider the fact that a number of medical conditions are associated with autism, such as seizures, blindness, or gastrointestinal diseases. Identifying accurately whether the symptoms are secondary to another medical condition or represent the exacerbation of pre-existing ASD may have implications for both immediate management and prognosis.^39^ Table 2 summarizes the medical evaluation procedures recommended for ASD.

**Table 2:** Medical evaluation procedures for autism spectrum disorder

Purpose	Procedures
• Useful to clarify risk factors, guide future investigations, and identify and treat comorbidities	prenatal, perinatal, and family medical history physical examination: growth parameters (e.g., height, weight, and head circumference), skin examination (e.g., for tuberous sclerosis complex or neurofibromatosis), neurological examination, and assessment of dysmorphisms
• Useful to clarify differential diagnosis and provide adequate support and interventions	hearing and vision assessment
• Useful to assess the genetic etiology of ASD, predict recurrence, treat co-occurring conditions, and avoid further unnecessary testing	genetic testing: depending on jurisdiction, all individuals with ASD or only those with intellectual disability, dysmorphic features, or congenital anomalies.
• Rule out epilepsy, Landau-Kleffner syndrome, and electrical status epilepticus of sleep	electroencephalography (prolonged or with sleep record preferred), especially in individuals with seizures or late or atypical regression
• Identify neurological conditions that provide etiological insights and often require monitoring and treatment	structural brain MRI: individuals with atypical regression, dysmorphology, microcephaly, macrocephaly, seizures, severe intellectual disability, focal neurological findings, severe hypotonia or muscle weakness, and other relevant clinical indicators
• Identify metabolic disorders associated with autism spectrum disorder that can be treatable. Differential diagnosis may also be indicated	blood and urine metabolic testing: individuals with cyclic vomiting, lethargy with minor illnesses, atypical regression, seizures, and other relevant clinical indicators
• Identify pica increases the risk for lead intoxication	blood levels for lead: individuals with pica or known exposure to lead (no evidence in favor of routine testing of hair, blood, or urine for environmental toxins or heavy metals)

Modified from Lord et al.^30^

Beyond diagnostic accuracy, a diagnosis of ASD is certainly a significant event in any stage of life for individuals and their families; therefore, it is essential that clinicians provide meaningful information about the diagnosis and prognosis to improve treatment planning and quality of life.

Although clinical heterogeneity remains a critical obstacle in the development of reliable diagnostic criteria in autism, common efforts in novel areas of investigation may help refine the diagnostic process and assist in the identification of subsets of autistic individuals favoring early identification, targeted interventions, and personalized medicine approaches. These ongoing efforts are discussed in the next sections.

## Perspectives on genetic assessment

**Figure 1:**
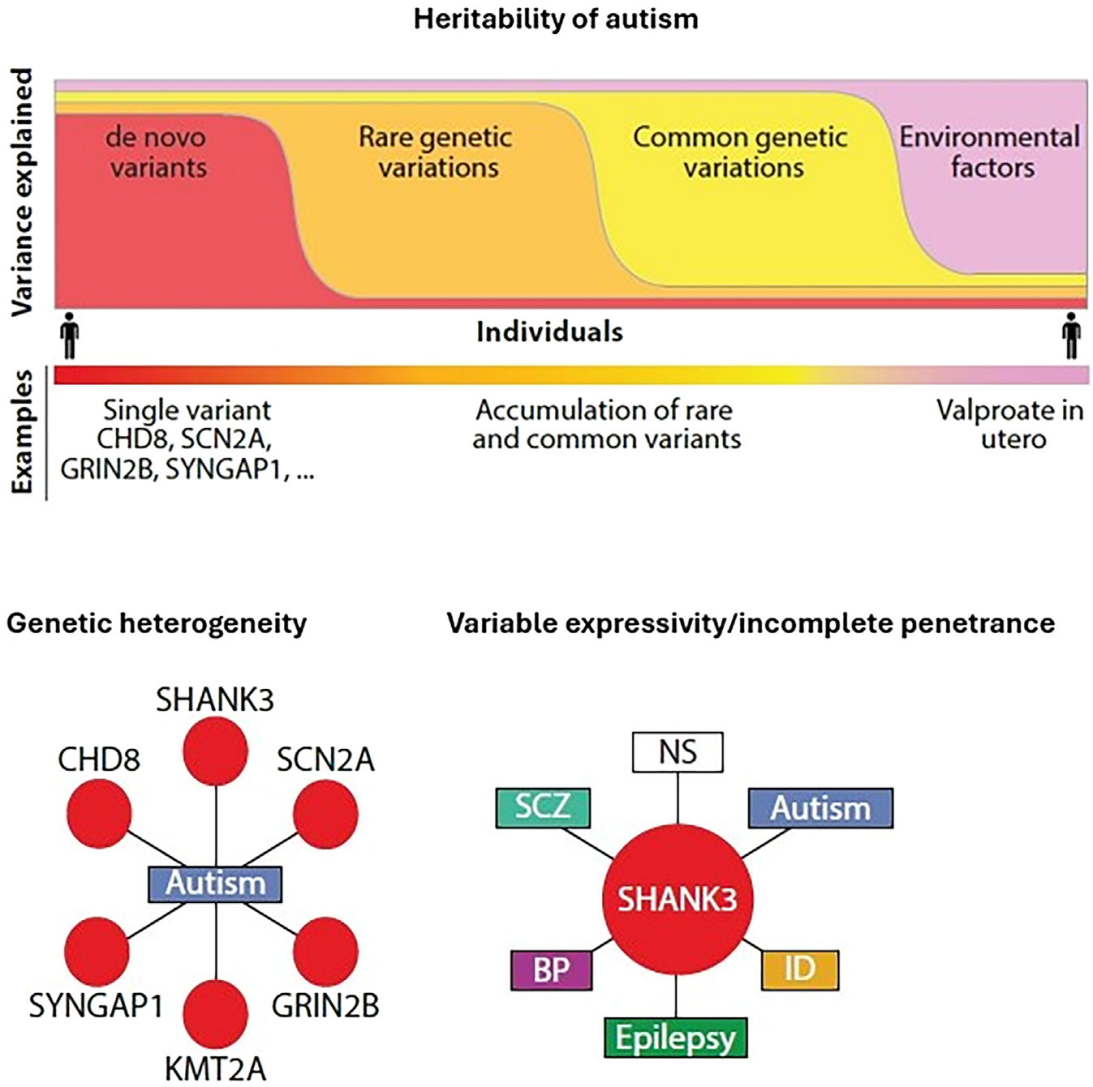
Heritability, genetic heterogeneity, and variable expressivity/incomplete penetrance in ASD Abbreviations: BP, bipolar disorder; ID, intellectual disability; NS, no symptoms; SCZ, schizophrenia. Reproduced, with permission, from Leblond et al.^40^

Variation in autistic traits is influenced by a combination of *de novo* mutations, rare inherited variants, common inherited variants, and environmental factors. Genetic variants in different genes can contribute to ASD (heterogeneity), while variants within the same gene may be linked to a range of co-occurring symptoms or, in some cases, no symptoms at all (variable expressivity/incomplete penetrance) (Figure 1).

Genetics ought to be part of the diagnostic assessment of all individuals with ASD, contingent upon accessibility of technology and affordability of costs. The reason behind genetic testing for ASD is not to provide an “autism” diagnosis, which is based on formal criteria such as those in the DSM-5-TR or ICD-11, but rather to provide information on specific etiologic factors or genetic contributions underlying the phenotypic behavioral alterations. The state-of-the-art assessment varies greatly according to new technological and methodological advances and their costs. Currently, the genetic assessment in public healthcare systems commonly includes array-based cytogenetics as first tier, employing either single-nucleotide polymorphism (SNP) or comparative genomic hybridization (CGH) arrays, and the exploration of the whole exome based on whole-exome sequencing (WES) as second tier diagnostic testing (see Figure S1).^41^ In general, the most cost-effective strategy followed by national healthcare systems is to request WES after SNP array results are negative.^42^ Importantly, many pathogenic copy-number variants (CNVs) associated with ASD are relatively small in size, so array technology for clinical use in neurodevelopmental disorders (NDDs), including ASD, needs to have sufficient sensitivity (at least 50 kb or less). In addition, karyotyping and fragile X testing also remain highly recommended: the former for large chromosomal abnormalities and balanced translocations, as well as for mosaicism (see below); the latter since the genotype-phenotype correlation in fragile X syndrome is rather weak.^42^ More specific genetic and/or metabolic tests may be sought for autistic individuals with a medical history suggesting a syndromic form of NDD. If this general protocol is applied, the probability of detecting “certainly pathogenic” or “probably pathogenic” variants largely depends on the severity of the clinical picture and on the presence of co-morbid intellectual disability (ID) or seizures. Briefly, the yield of pathogenic variants obtained with SNP array and WES averages 8.1% and 15.0%, respectively, in ASD samples, but goes up to 13.7% and 37% in samples with ASD and co-morbid ID.^41^ Whole-genome sequencing (WGS), which is mainly used for research purposes, may change this yield to a significant extent since it more reliably examines genomic regions of the exome that are particularly difficult to sequence using standard WES (such as “CG”-rich regions of *SHANK3*). If this genetic diagnostic protocol is followed, “certainly pathogenic” or “probably pathogenic” variants are detected on average in 23.5% of ASD samples and in 52%–53% of samples with ASD and co-morbid ID.^42^ To date, this yield is by far the largest provided by any medical test performed in NDDs.

Despite this sizable percentage of genetic positives, two major drawbacks still remain. First, once a “certainly pathogenic” or “probably pathogenic” variant is detected, only in a minority of individuals does this information significantly influence clinical management.^43^ Putative examples of actionable genomics in clinical practice include increased prognostic predictive power conferred by genetic testing, the correct interpretation of the appearance of an infrequent sign/symptom, a more appropriate recommendation for medical tests and scans, and possibly even a specific psychopharmacological or behavioral intervention.^44^ However, these examples are still limited in clinical practice, and much more cross-talk is needed between basic neurobiology, genetics, and child psychiatry to transfer the knowledge derived from genetic testing into better clinical management. Second, even the most thorough genetic testing strategy yields negative results in the majority of individuals with ASD.

Somatic mosaicism and abnormal epigenetics are two mechanisms that could contribute to these genetically negative cases. Somatic mosaicism, due to mutations such as single-nucleotide variants (SNVs) and CNVs, can occur in any tissue, including the brain.^45^ Approximately 0.8%–1.3% of autistic probands carry a mosaic deleterious (i.e., that increases an individual’s susceptibility or predisposition to ASD) SNV/CNV affecting genes potentially related to ASD risk.^46^ Somatic deleterious SNV/CNV that occur early enough in development to be detectable in blood-derived DNA may explain as many as 5% of cases of ASD.^47^ Otherwise, detection of brain-selective mosaicism may require deep sequencing of DNA extracted from brain tissue,^48^ which further limits the clinical applicability of this approach.

Epigenetic variations can profoundly affect gene expression by modifying the chromatin structures and can impact the DNA reading frame of genes associated with ASD.^49^ Importantly, epigenetic signatures have been found not only to differentiate autistic and typically developing individuals following diagnosis,^50^ but also at birth in DNA extracted from cord blood,^51^ and even prenatally in DNA extracted from the sperm cells of fathers of autistic children.^52^ This clearly poses major questions on the functional relevance of these epigenetic variants and, most importantly, on transgenerational contributions to the pathophysiology of ASD.^53^

Finally, common genetic variants increasing ASD risk and contributing to build a “polygenic risk score” (PRS) for ASD appear especially enriched in methylation sites, pointing to these common genetic variants as an unexpected cross-road between genetic predisposition and epigenetic mechanisms.^54^ In the era of genome-wide association studies (GWASs), the translation of PRS into the clinic raises increasing interest, but currently inter-individual differences in ASD pathogenesis and inter-ethnic differences in population structure (i.e., linkage disequilibrium) represent a major obstacle to the use of PRS in clinical setting.^54^

In conclusion, a complete panel of genetic tests for ASD, including karyotyping, fragile X, SNP-CGH, and WES (WGS), provides a positive result in up to about 50% of cases, depending on autism severity and comorbidity with ID. By comparison, brain MRI provides a positive result in only 7.2% of patients with ASD and typically produces no therapeutic benefit. By providing etiologic clues and information on genetic contributions to behavioral symptoms, knowledge derived from genetic testing can relieve parents from the burden of not knowing what caused ASD in their children, can unveil genetic syndromes whose characteristics and clinical course may already be well known, and in some cases can promote better clinical management. At the same time, genetic contributions represent a conundrum that is unlikely to provide a satisfactory explanation for ASD in any individual, if genetics remains the only level of analysis. Instead, a panel encompassing biomarkers from multiple levels of analysis and including, but not limited to, genetic variants (pathogenic or at risk) will more likely be able to capture the complexity of ASD genetic architecture and to dissect autism into subgroups with relatively homogeneous pathogenetic underpinnings and, hopefully, meaningful clinical implications.^55^ Overall, genetic information is not yet diagnostic but provides valuable background information to support clinical care and personalized interventions.

## Perspectives on telemedicine

Although most diagnostic assessment procedures, including the genetic evaluation discussed in the previous section, have been devised to be carried out face-to-face, the limited availability of services has prompted the development of remote assessment, and this trend has been further enhanced by the impact of the COVID-19 pandemic. ASD screening or assessment could involve telemedicine, namely the use of digital technology to connect providers with patients or their caregivers when they are separated by distance.^56^ Telemedicine for screening and assessment of ASD can be classified based on (1) the type of information transmitted (e.g., text, audio, and video), (2) the device used (e.g., computer, tablet, and smartphone),^57^^,^^58^^,^^59^^,^^60^ and (3) the different timing of the information transfer, i.e., synchronous or asynchronous. Synchronous or real-time methods require live interactions and/or observations conducted via video-conferencing services.^61^^,^^62^^,^^63^^,^^64^^,^^65^^,^^66^^,^^67^ In contrast, asynchronous or store-and-forward methods involve questionnaires being completed^58^^,^^60^^,^^68^^,^^69^^,^^70^^,^^71^ and relevant video recordings of live events of individuals with ASD being collected by caregivers and then forwarded to a clinician for further evaluation.^72^^,^^73^ There is evidence of high agreement between diagnosis made via telemedicine and in-person assessments.^74^

The use of telemedicine has been proven helpful for performing initial ASD screening, speeding up the assessment process, reducing the time required for the diagnosis, and ensuring faster access to appropriate therapies, albeit with some limitations.^75^^,^^76^ In fact, telemedicine makes it possible to reduce distances, save time and costs, and observe the patient’s spontaneous behavior and natural expressions in the home environment. However, telemedicine requires a few prerequisites that are not yet evenly distributed across the clinical population, such as the availability of valid information technology equipment, sufficient familiarity with the technology, and a fast internet connection. In addition, simply observing individuals with ASD in a predictable and familiar environment could mask some of their dysfunctional behaviors. Therefore, although several studies have investigated the accuracy, validity, and feasibility of telematic assessment for ASD with promising results,^77^^,^^78^ telemedicine is now seen as a complement to traditional face-to-face clinical assessment rather than an exclusive alternative. Table S2 summarizes (when available) the sensitivity, specificity, area under the curve (AUC), positive predictive value (PPV), and negative predictive value (NPV) of tools delivered via telemedicine that have been assessed in terms of supporting the diagnostic process of ASD.

## Digital technologies in clinical practice

Research on digital technologies in ASD is a promising field for supporting early recognition, precision diagnosis development, and personalized prognostic and treatment strategies, providing objective and operator-independent data. Indeed, digital technologies have the advantage of making clinical decisions more objective, reliable, and evidence-based, while reducing clinical resources and waiting times for diagnostic assessment.^79^ Automated video analysis, sensors and wearables, and virtual reality are the most commonly investigated diagnostic digital technologies for ASD. In addition, mobile apps and software able to integrate information from multiple sources (with or without questionnaires filled by caregivers or health professionals) have been studied.^80^^,^^81^^,^^82^^,^^83^^,^^84^^,^^85^

However, currently digital tools are used mainly for research purposes to detect and study candidate cognitive, behavioral, and peripheral physiological diagnostic markers of ASD. Within the cognitive domain, executive functions and attention skills are the most explored functions, along with other cognitive constructs including “cognitive load in learning complex tasks,” such as driving.^86^ These constructs can be studied using digital adaption of traditional neuropsychological tests (e.g., Tower of London test)^87^ or integrating multiple information (e.g., pupil dilations and electroencephalogram [EEG] data to track cognitive and attentional load).^88^ In relation to behavioral domains, digital tools can automatically detect peculiarities in verbal behaviors, particularly prosody or idiosyncratic utterances,^89^ as well as vocalizations^90^ or speech and turn-taking parameters,^91^ and non-verbal behaviors. For these purposes, eye tracking is the most used tool, since it allows one to study and measure eye movements and direct gaze non-invasively, making it suitable even for toddlers or infants with suspected ASD. Indeed, it has been demonstrated that eye tracking can reveal different gaze patterns associated with ASD, such as pronounced preference toward geometric figures than social images in infants and toddlers with autism,^92^^,^^93^ different fixation patterns on social stimuli, as well as atypical gaze behaviors related to deficit of joint attention.^94^

In addition, reduced abilities in gross and fine motor skills, as well as atypical motor pattern or lower motion complexity, can be identified in children with ASD through video-analysis motion tracking technologies, motor sensors applied on objects with which the child interacts, and wearables.^95^^,^^96^^,^^97^ Moreover, as a new field of research, automated video-analysis technology has been used to quantify “social synchrony.” This is defined as *the alignment of an individual’s own behaviors* (*intrapersonal synchrony*) *and the reciprocal coordination of others’ behaviors* (*interpersonal synchrony*)*.* These behaviors are coordinated either simultaneously or in specific temporal sequence patterns, demonstrating its reduction in individuals with ASD.^98^^,^^99^^,^^100^ Lastly, peripheral physiological variations, such as heart rate, EEG signals, or electrodermal activity, can be revealed by wearables to categorize the autonomic nervous system responses in individuals with ASD during various tasks (e.g., joint attention or emotion recognition tasks).^101^

**Figure 2:**
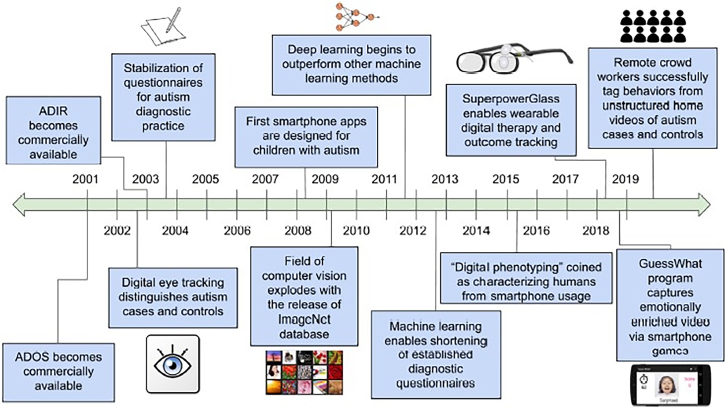
Timeline of critical events for the field of digital autism diagnostics Abbreviations: ADIR, Autism Diagnostic Interview–Revised; ADOS, Autism Diagnostic Observation Schedule. Reproduced, with permission, from Washington et al.^103^

Considered as a whole, digital diagnostics (in particular, those developed to assess behavioral markers) are generally not invasive, and this favors their use with high-sensitive individuals such as those with autism. Digital tools can capture details that are otherwise imperceptible to the human eye (e.g., eye tracking for fixing the gaze on social stimuli has a very high detection frequency) or in any case difficult to detect or moreover quantify. Data reported in individual studies concerning diagnostic accuracy digital tools are promising (Table S2). Although these tools are not yet used in practice to support the diagnosis of autism, some of them, such as eye tracking, which has generally fairly high test-retest reliability (e.g., for attentional bias^102^), may eventually be implemented in clinical practice. However, to date, specific recommendations for the development and validation of digital diagnostics are still lacking. Figure 2 summarizes the timeline of key events in the field of digital autism diagnostics.

## ML and AI

**Figure 3:**
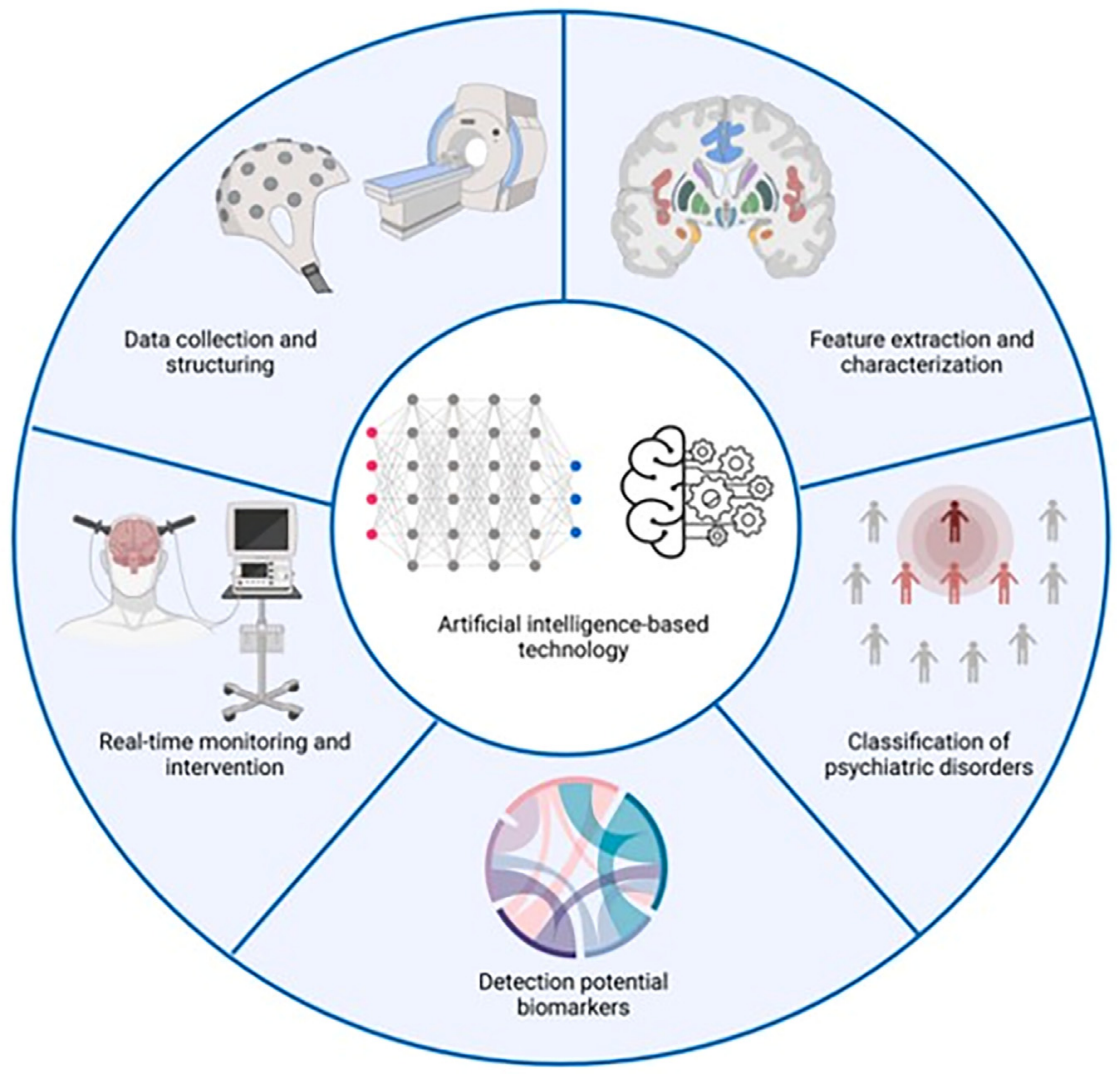
Possible implications of artificial intelligence-based technology for the diagnosis and management of mental and neurodevelopmental conditions, including ASD Reproduced, with permission, from Sun et al.^105^

The implementation of digital technologies in the clinic could be supported by ML and AI, which have recently started to play an important role in the screening/diagnosis and understanding of mental and neurodevelopmental conditions including ASD^104^ (Figure 3). ML is a subfield of AI, more specifically an approach used to simulate intelligent human behavior when analyzing complex and large datasets to recognize specific patterns. In brief, ML models develop innovative algorithms and statistical models by analyzing and getting *trained* on large sets of data, from which patterns are recognized and new predictions or decisions are made. Within the discipline of computational psychiatry, ML is used to recognize patterns in data (e.g., neuroimaging or electrophysiological data, large electronic health record datasets), classify cases into categories (e.g., investigating if different clinical groups can be differentiated based on clinical or neuropsychological data), and make predictions about prognostic or interventional outcomes.

In the last few years, the use of ML to support the diagnosis and understanding of ASD has been extensively investigated.^106^^,^^107^^,^^108^^,^^109^^,^^110^^,^^111^^,^^112^ While ML cannot be used *during* screening and/or diagnosis of ASD, it can be used to identify patterns directly associated with ASD. These patterns—upon proper testing and validation—could be implemented as objective diagnostic biomarkers and used to confirm a clinical (but subjective) diagnosis of ASD, partly overcoming the limitations of the current diagnostic procedures.

In relation to early screening and diagnosis of ASD, ML approaches have been often used to understand if early evaluations of general infant behavior (e.g., parent-rated) predict a formal diagnosis of ASD at a later age. For example, an ML model based on parent-rated early learning and adaptive functioning at 14 months was able to predict a formal diagnosis of ASD at 3 years with moderate accuracy.^113^ Home video recordings can also be used to train ML algorithms to identify behavioral patterns that discriminate between autistic and non-autistic individuals.^114^ Other studies implemented ML models to analyze motor features and development. For example, Crippa et al.^115^ found that preschoolers with ASD could be distinguished from their typically developing peers based on differences in goal-oriented movements (e.g., transporting an object to a target area).

ML and computer vision approaches have also been used to document and quantify signs related to the visual system during infancy that are associated with ASD diagnosis later in life, such as atypical visual attention or non-smooth visual tracking^116^ or subtle abnormalities in producing and recognizing emotions in pattern of facial expressions.^117^ Analysis of eye-tracking data via ML/AI approaches may be potentially helpful to identify individuals with ASD with high accuracy, especially in preschool-aged children.^112^ Nevertheless, studies that investigated ML approaches for ASD early diagnosis showed that sensitivity, specificity, and accuracy varied from 0.50 (poor discrimination between individuals with and without ASD) to 1.00 (excellent discrimination),^118^ highlighting the need for further rigorous and larger studies.

Other studies have shown that ML can help to simplify the assessment process, e.g., by identifying the essential items in questionnaire, interviews, or behavioral assessment that need to be retained without undermining diagnostic accuracy. For example, ML-based studies showed that a lower number of activities/items in the different modules of ADOS and ADI could be sufficient to diagnose ASD and be as accurate as the full and time-consuming assessments.^119^ However, and crucially, many behavioral studies in the field of ML are hampered by analytic limitations in terms of lack of independent dataset for the external validation or lack of use of appropriate validation methods such as k-fold cross-validation.^120^

One of the most prominent data modality used in ML/AI is neuroimaging. ML could help identify subtle brain structural and functional differences between autistic and non-autistic individuals, integrating information from multiple sources.^107^ According to recent systematic reviews and meta-analyses,^121^ ML and deep learning algorithms are relatively accurate in discriminating between autistic and non-autistic individuals, through neuroimaging data. For example, structural and functional MRI-based ML algorithms showed moderate to good sensitivity and specificity for discriminating between individuals with and without ASD, but replication is often lacking.^122^

Another field of application of ML in supporting ASD diagnosis is genomics. Based on widespread shared open-access genetic datasets, ML could be helpful for identifying new genetic markers of ASD^123^ or supporting diagnostic screening for ASD based on genetic variability.^111^ Furthermore, exploration of large healthcare databases using ML approaches has enhanced our ability to identify ASD-specific electrophysiological^124^ or blood-based biomarkers,^125^ allowing an improved understanding of ASD heterogeneity.^126^ For example, ML applied to electroencephalography and magnetoencephalography data can help classify and predict ASD diagnosis in high-risk infants at 3 months of age and predict symptom severity, with high accuracy.^108^ A recent systematic review retrieved 27 relevant studies to date.^108^ Indeed, in a meta-analysis of 232 studies using AI based on overall nine modalities, the accuracy based on of the EEG data was the best, with AUC = 0.89 (95% CI: 0.85–0.93). AUC for other relevant modalities were as follows, in descending order of magnitude: eye tracking = 0.83 (0.76–0.93), task-based functional MRI = 0.79 (0.75–0.83), resting-state functional MRI = 0.74 (0.72–0.76), diffusion weighted and tensor imaging = 0.74 (0.69–0.80), structural MRI = 0.73 (0.65–0.79), and multimodal = 0.71 (0.59–0.80).^127^ Lastly, ML/AI have been applied to other data streams such as digitized historical health records, voice, motion, and other behavioral features,^128^ questionnaires, sociodemographic, familial, and environmental data.^104^

Overall, although ML/AI approaches represent a promising tool to unveil complex mechanisms underlying behavioral and emotional patterns in autism, implementation in clinical practice remains challenging. First, large datasets are required to train ML algorithms and test them. In some cases, this is achieved by combining datasets from different studies or public repositories, which however increases data heterogeneity. Using large datasets to make speculations (or conclusions) about the whole population of autistic individuals also collides with the widely acknowledged idea that autism is heterogeneous and can present in different individuals with different and wide ranges of symptoms and features. Furthermore, considering only specific features that are thought to be associated with ASD can overlook individual features and needs associated with disorders and conditions that co-occur with ASD. Finally, in particular for neuroimaging-based tools, the costs, which still remain too high to be implemented in publicly funded healthcare systems, and the difficulties in scanning uncooperative children—with the possible exception of resting-state MRI during sleep—are important challenges.

Despite such limitations, research on ML/AI methods for screening/diagnosis of ASD has also led to the development of software and devices that are currently implemented in some clinical contexts. For instance, the Food and Drug Administration (FDA)-approved Canvas Dx (https://cognoa.com/) implements ML algorithms on data received by parents/caregivers (e.g., questionnaires and home videos), video analysts, and healthcare professionals and informs about a possible diagnosis of ASD. Canvas Dx demonstrated excellent sensitivity (98.4%) and good specificity (78.9%) among participants for which the tool was able to make a decision (<50% of the sample).^83^ This makes Canvas Dx a good example of promising applications of ML/AI methods for supporting the diagnostic assessment of ASD. Table S3 summarizes (when available) the sensitivity, specificity, AUC, PPV, and NPV of digital/ML tools that have been assessed in terms of supporting the diagnostic process of ASD.

## Putative candidate diagnostic biomarkers

A biomarker is defined by the FDA National Institute of Health Biomarker Working Group (US) as “an indicator of normal biological processes, pathogenic processes, or biological responses to an exposure or intervention.”^129^

A biomarker needs to be sensitive, accurately identifying as positive those individuals who have the outcome of interest, and specific, accurately labeling as negative those individuals who do not have the outcome of interest. Although there are no established benchmarks for these metrics, quantitative measures that enable diagnostic accuracy with at least 80% sensitivity and 80% specificity are often considered clinically useful.^130^ The American Psychiatric Association Work Group on Neuroimaging Markers of Psychiatric Disorders suggested that a promising biomarker should have two or more independent well-powered studies providing evidence of sensitivity and specificity at least of 80%.^131^ In addition, a biomarker would need to have good PPV, NPV, internal validity, be externally valid, and be reliable in terms of test-retest reliability and inter-rater reliability.

The largest systematic review of candidate diagnostic biomarkers in NDDs, including ASD, assessed a wide range of potential genetic, biochemical, neuroimaging, neurophysiological, and neuropsychological measures.^132^ Among these, biochemical markers have been the most investigated, with 300 studies identified and a total of 1,289 biochemical measures tested. However, only 73 measures were reported by at least two studies with at least one positive finding and more than 50% replications. Among those with only positive replications in the same direction, the most replicated were coproporphyrin (a product of heme synthesis, increased), glutamine (decreased), 8-isoprostane (a prostaglandin isomer, increased), cysteine (decreased), glutathione/oxidized glutathione ratio (decreased), lead (increased), neurotensin (increased), 4-methylphenol (a phenol derivative, increased), secreted amyloid precursor protein alpha (a neurotrophic protein, increased), succinic acid (increased), and human transforming growth factor β (increased). Highest specificity and/or sensitivity were achieved by oxytocin (decreased), vitamin E (decreased), interferon-gamma-inducible protein-16 (increased), interferon-gamma (increased), and heat shock protein 70 (increased). However, none of these measures met the criteria to be identified as a biomarker (all the references for relevant studies on these compounds are freely available in supplemental material 1 and supplemental Table 5 accompanying the main text of Cortese et al.,^132^ https://osf.io/wp4je/?view_only=8c349f45a9ac441490981acf946c8d9a).

Considering common genetic variants, this systematic review identified only a GWAS specifically aiming at identifying SNPs in ASD.^133^ This included over 18,000 individuals with ASD and almost 28,000 neurotypical controls and identified five SNPs significantly associated with ASD. The corresponding candidate genes have been previously involved in neuronal function and neurodevelopment. For instance, these included PTBP2, which encodes for a splicing regulator; CADPS, encoding a calcium-binding protein involved in neurotransmission; and KCNN2, which encodes for a voltage-independent Ca^2+^-activated K^+^ channel and thus is involved in neuronal excitability. The estimated SNP-based heritability (SNP-h^2^) for ASD was 11.8%. Overall, this GWAS was well conducted and provided valuable knowledge on the genetic underpinning of ASD. However, it did not provide metrics, such as sensitivity and specificity, needed to assess the identified loci as diagnostic biomarkers.

Several neuroimaging studies have compared brain characteristics between autistic individuals and controls from the general population. However, most of them aimed at investigating the neurobiology of ASD, rather than identifying potential imaging biomarkers. Among the 115 neuroimaging studies identified by Cortese et al.,^132^ 47% reported only *p* values and no other metrics needed to define a biomarker.

Among neurophysiological measures, only the acoustic eyeblink startle latency was consistently replicated among three studies (increased in ASD) (for references, please supplemental material 1 and supplemental Table 12 accompanying the main text of Cortese et al.,^132^ https://osf.io/wp4je/?view_only=8c349f45a9ac441490981acf946c8d9a). Finally, considering neuropsychological tests, only long-term and short-term memory measures were replicated across a small number of studies (two and five respectively) (for references, please Supplemental Material 1 and Supplemental Table 15 accompanying the main text of Cortese et al.,^132^ https://osf.io/wp4je/?view_only=8c349f45a9ac441490981acf946c8d9a). Notably, these measures obtained 100% replication in both studies in ASD and ADHD samples, which supports their transdiagnostic nature. However, both neurophysiological and neuropsychological studies did not consistently provide metrics necessary to assess the identified measures as diagnostic biomarkers.

Overall, the systematic review by Cortese et al.^132^ highlighted that, despite the large number of studies and measures considered, to date, there are no metrics that meet the criteria for a diagnostic biomarker. This lack of replicable findings can be both explained by challenges inherent in the search for biomarkers, especially for neurodevelopmental conditions, and by methodological limitations. Clinical presentation, neuropsychological profiles, and comorbidities vary greatly in ASD. Most studies to date included small samples and were thus underpowered to stratify individuals into more clinically and biologically homogeneous subgroups, which may help identify suitable biomarkers. Methodological limitations, such as lack of standardization, confounding factors, and limited replicability, have also hampered progress in the field. Heterogeneity in terms of laboratory procedures, imaging methods, and analysis techniques can also affect comparability among studies for external validation and replicability. Most studies focused on associations and reported *p* values, which are poorly informative. Finally, once a measure has been identified, the biological significance often remains to be elucidated. For instance, considering biochemistry, vitamin E and inflammatory markers were the most replicated, but may be related to diet or stress response rather than ASD itself.

### Conclusions

In current clinical practice, the diagnosis of ASD still primarily relies on clinical judgment aided by questionnaires and structured interviews/observation. The diagnostic process can be complex, especially in the presence of co-occurring conditions, very young age, or less typical presentations. It may also be time-consuming and expensive for clinical services. Thus, in addition to alternative diagnostic pathways such as those that involve partnerships with community providers,^134^ there have been increasing efforts to identify approaches and tools that can assist this process by providing more objective and accurate measures. This may be particularly important for ASD, given its highly heterogeneous presentation, and may guide the identification of the individual needs and thus a more tailored support. Notably, current diagnostic tools are highly valuable but have mainly been developed for males and may not fully capture the nuances of the ASD presentation in females. This may lead to delayed recognition and less effective therapeutic interventions for secondary presentations, such as anxiety or depression in adolescence. Thus, improving the ability of diagnostic tools to capture gender differences warrants further investigation.

To date, no tools can replace or promise to replace the clinical diagnostic assessment. Genetic testing may contribute to the diagnostic process especially in cases of comorbid ID or seizure. When a thorough genetic diagnostic protocol is followed, pathogenic variants can be detected in up to 23.5% of ASD samples and in 52%–53% of samples with ASD and co-morbid ID. This is the largest yield provided by a medical test for neurodevelopmental disorders to date. Nevertheless, it is important not to disregard negative results as not all variants associated with ASD are known or can be accurately detected. Moving forward, it will be important to strengthen the link between these investigations and clinical practice, especially as they can potentially guide more tailored management approaches.

Digital diagnostics are emerging as promising tools as they are generally not invasive and able to capture subtle variations in behavior, such as in eye movements, that would otherwise be difficult to capture clinically. Nevertheless, future larger and more rigorous studies are needed to refine the diagnostic accuracy of these approaches and their potential clinical applications. Similarly, AI/ML approaches have been tested on a range of data, including behavioral, neuropsychological, and neuroimaging data. These approaches offer the advantage of combining multi-level data and may help understand the biological correlates of the observed phenotypes. However, their applicability may be limited, especially for neuroimaging, in particular due to the high cost. Nevertheless, cost-effectiveness, rather than simply costs, may need to be considered moving forward and should be investigated when assessing new tools.

Crucially, to date, there are no metrics that meet the criteria for a diagnostic biomarker. Beyond challenges related to the heterogeneity of ASD, progress in the field has been hampered by methodological limitations, including small samples, lack of standardization, and limited replicability. Going forward, further international collaborations may support larger and more robustly designed studies and help develop multimodal datasets to combine biomarkers, thus enhancing accuracy and ensuring reproducibility as well as meaningful clinical translation.

## Acknowledgments

S.C., NIHR Research Professor (NIHR303122), is funded by the NIHR for this research project. The views expressed in this publication are those of the author(s) and not necessarily those of the NIHR, NHS, or the UK Department of Health and Social Care. S.C. is also supported by NIHR grants NIHR203684, NIHR203035, NIHR130077, NIHR128472, and RP-PG-0618-20003 and grant 101095568-HORIZONHLTH- 2022-DISEASE-07-03 from the 10.13039/100020668European Research Executive Agency.

## Declaration of interests

S.C. has declared reimbursement for travel and accommodation expenses from the Association for Child and Adolescent Central Health (ACAMH) in relation to lectures delivered for ACAMH, the Canadian ADHD Alliance Resource, and the British Association of Psychopharmacology and from Healthcare Convention for educational activity on ADHD, and S.C. has received honoraria from Medice.

A.M.P. has been a consultant to and/or speaker for and has received honoraria from Servier, Sanofi, and Healx Limited.

M.S. has received honoraria/has been a consultant for AbbVie, Angelini, Lundbeck, and Otsuka.

P.F.-P. has received research fees from Lundbeck and received honoraria from Lundbeck, Angelini, Menarini, and Boehringer Ingelheim.

## Supplemental information

Document S1. Figure S1 and Tables S1–S3

Document S2. Article plus supplemental information
