# Supplementary material for: Latest clinical frontiers related to autism diagnostic strategies
Source: Cell Rep Med. 2025 Jan 28;6(2):101916. doi: 10.1016/j.xcrm.2024.101916 (PMC11866554; doi:10.1016/j.xcrm.2024.101916)
Supplement: Document S1. Figure S1 and Tables S1–S3 [file mmc1.pdf]

Cell Reports Medicine, Volume *6*

Supplemental information

Latest clinical frontiers related

to autism diagnostic strategies

Samuele Cortese, Alessio Bellato, Alessandra Gabellone, Lucia Marzulli, Emilia Matera, Valeria Parlatini, Maria Giuseppina Petruzzelli, Antonio M. Persico, Richard Delorme, Paolo Fusar-Poli, Corentin J. Gosling, Marco Solmi, and Lucia Margari

Supplement to Cortese et al., "Latest clinical frontiers related to autism diagnostic strategies"

Table S1. Summary of diagnostic criteria for Autism Spectrum Disorder. 2

Figure S1. 3

Table S2. Performance of telemedicine tools in Autism Spectrum Disorder. 4

Table S3. Performance of digital/ML tools in Autism Spectrum Disorder. 5

Table S1. Summary of diagnostic criteria for Autism Spectrum Disorder.

DSM-5-TR	ICD-11
**A.** Persistent deficits in social communication and social interaction across multiple contexts, as manifested by all of the following, currently or by history:<br>• Deficits in social-emotional reciprocity<br>• Deficits in nonverbal communicative behaviors used for social interaction<br>• Deficits in developing, maintaining, and understanding relationships<br><br>**B.** Restricted, repetitive patterns of behavior, interests, or activities, as manifested by at least two of the following, currently or by history:<br>• Stereotyped or repetitive motor movements, use of objects, or speech<br>• Insistence on sameness, inflexible adherence to routines, or ritualized patterns of verbal or nonverbal behavior<br>• Highly restricted, fixated interests that are abnormal in intensity or focus<br>• Hyper- or hypo-reactivity to sensory input or unusual interest in sensory aspects of the environment<br><br>**C.** Symptoms must be present in the early developmental period<br><br>**D.** Symptoms cause clinically significant impairment in current functioning<br><br>**E.** Disturbances are not better explained by intellectual developmental disorder or global developmental delay	**Essential (Required) Features**<br>Persistent deficits in initiating and sustaining social communication and reciprocal social interactions that are outside the expected range of typical functioning given the individual's age and level of intellectual development. Specific manifestations of these deficits vary according to chronological age, verbal and intellectual ability, and disorder severity.<br><br>Persistent restricted, repetitive, and inflexible patterns of behavior, interests, or activities that are clearly atypical or excessive for the individual's age and sociocultural context.<br><br>The onset of the disorder occurs during the developmental period, typically in early childhood, but characteristic symptoms may not become fully manifest until later, when social demands exceed limited capacities.<br><br>The symptoms result in significant impairment in personal, family, social, educational, occupational or other important areas of functioning.<br><br>Some individuals with Autism Spectrum Disorder are able to function adequately in many contexts through exceptional effort, such that their deficits may not be apparent to others. A diagnosis of Autism Spectrum Disorder is still appropriate in such cases.
**Specifiers:**<br>• Current severity of A and B based on requiring support<br>• Presence/absence of accompanying intellectual impairment<br>• Presence/absence of accompanying intellectual impairment<br>• Associated with known genetic or other medical condition or environmental factor<br>• Associated with a neurodevelopmental, mental, or behavioral problem<br>• With catatonia	**Specifiers:**<br>• Co-occurring Disorder of Intellectual Development (presence/absence)<br>• Degree of Functional Language Impairment<br>• Loss of Previously Acquired Skills<br>• Other Specified Autism Spectrum Disorder<br>• Autism Spectrum Disorder, Unspecified

Figure S1. Genetic testing of individuals with ASD

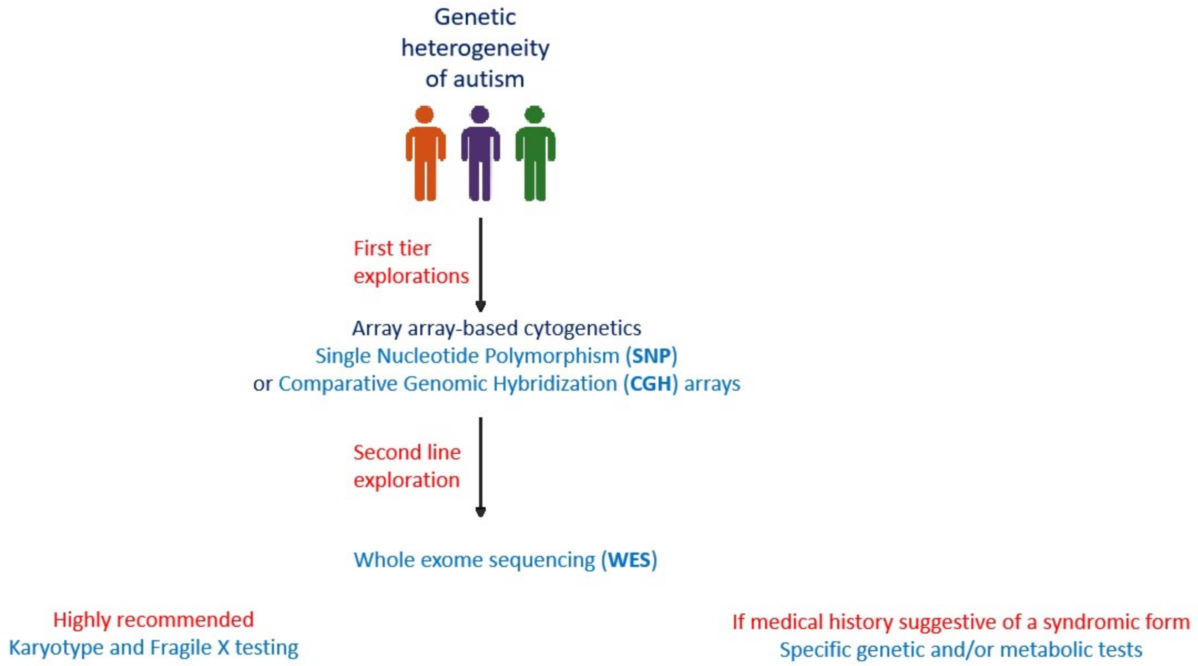

Legend: Genetic testing in ASD

Table S2. Performance of telemedicine tools in Autism Spectrum Disorder.

First author, year	Telemedicine tool	sensitivity	specificity	AUC	PPV	NPV
Ben-Sasson, 2018 [65]	ML applied to web-source completion of the Modified Checklist for Autism in Toddlers-Revised, with Follow-Up and the Ages and Stages Questionnaire	NS	NS	74-88	NS	NS
Duda, 2016 [66]	Electronic screening tool *Mobile Autism Risk Assessment*	89.9	79.7	NS	67	95
Maleka, 2016 [56]	Smartphone application version of the Parents Evaluation Developmental Status tools	NS	NS	NS	100[a]	96[b]
Smith, 2017 [72]	*Tele-ASD-Peds*	96.9[c]	87.5[c]	NS	98.4[c]	77.8[c]
Sturner, 2016 [67]	Web-based Modified Checklist for Autism in Toddlers-Revised, with Follow-Up administered by primary care pediatrician	98	98	98[d]	98	NS

[a] positive correspondence with the paper-based tool

[b] negative correspondence with the paper-based tool

[c] scoring procedure with Likert scale

[d] accuracy

Reference numbers refer to the references in the main text

Table S3. Performance of digital/ML tools in Autism Spectrum Disorder.

First author, year	tool/technology	sensitivity	specificity	AUC	PPV	NPV
Al-Saei, 2023 [122]	ML applied to plasma protein glycation and oxidation	75	74	79	NS	NS
Anzulewic, 2016 [93]	ML analysis of the children's motor patterns during smart tablet gameplay:<br><br>Sharing Food game (threshold 0.50)	81	67	NS	NS	NS
	Sharing Food game (threshold 0.55)	76	73	NS	NS	NS
	Creativity game (threshold 0.50)	83	85	NS	NS	NS
	Creativity game (threshold 0.55)	80	88	NS	NS	NS
Bahado-Singh, 2019 [120]	ML applied to leucocyte epigenomic markers	97.5	100	95	NS	NS
Banerjee, 2023 [114]	ML Facial Expression Classifier on Mobile Devices	NS	NS	60.57[a]	NS	NS
Bussu, 2018 [110]	ML used to integrate multiple behavioural and developmental measures from multiple time-points for longitudinal ASD diagnosis prediction	60.7-69.6[b]	52.2-67.8[b]	65.1-71.3[b]	28.5-65.9[b]	66.4-91.5[b]
Crippa, 2015 [112]	ML applied to kinematic analysis of a simple reach-to-drop task (seven selected features)	100	93.8	96.7[a]	NS	NS
Li, 2019 [87]	hand-crafted feature based method as well as the end-to-end deep learning framework applied to atypical prosody (binary classification task)	100[c]	33.04[c]	NS	71.4[c]	100[c]
	hand-crafted feature based method as well as the end-to-end deep learning framework applied to stereotyped idiosyncratic phrases (binary classification task)	94.1[c]	50[c]	NS	88.9[c]	66.7[c]
Liu, 2016 [74]	ML applied to an eye movement dataset	93.10	86.21	89.63	NS	NS

	from a face recognition task					
Megerian, 2022 [81]	Canvas Dx ©	98.4%[d]	78.9%[d]	NS	80.8%[e]	98.3%[e]
Moon, 2019 [119]	ML applied to MRI	83	84	90	NS	NS
	ML applied to fMRI/Deep neural network	69	66	67	NS	NS
Pierce, 2010 [91]	eye-tracking in toddlers	NS	NS	68.6	100	NS
Pierce, 2016 [90]	eye-tracking (data here reported referred to the independent sample of 334 toddlers)	21	98	69	86	70
Plank, 2023 [89]	ML (SVM algorithm) applied to automated extraction of speech and interactional turn-taking	73.8	78.6	NS	NS	NS
Tariq, 2018 [111]	feature tagging of home videos for machine learning using mobile devices b	97	91	92[f]-94[f]	NS	NS
Wedyan, 2016 [95]	ML (SVM algorithm) applied to Upper Limb Motor Coordination. Throw part (non-accurate action)	75	73.33	NS	NS	NS
	ML (ELM algorithm) applied to Upper Limb Motor Coordination. Throw part (non-accurate action)	89.41	76	NS	NS	NS
	ML (SVM algorithm) applied to Upper Limb Motor Coordination.Fit part (accurate action)	74.12	73.33	NS	NS	NS

	ML (ELM algorithm) applied to Upper Limb Motor Coordination.Fit part (accurate action)	100	100	NS	NS	NS

AUC: area under curve; ELM: extreme learning machine; ML: machine learning; SVM: support vector machine.

[a] accuracy value used instead of AUC

[b] performance metrics are reported as range of minimum and maximum values obtained for the classifiers chosen as best (based on having the highest AUC) at different age (8 months or 14 months) and different sample grouping methods

[c] data calculated for the present work using the confusion matrix published along with the original paper

[d]For the 31.8% of participants who received a determinate output (ASD positive or negative)

[e]for all study completers

[f] results are here reported for the best ML classifier, LR5, in 4-6 y sample; for the AUC results are reported as a range: the minimum is the value found for all age groups, the maximum for children of age between 2 years and 6 years.

Reference numbers refer to the references in the main text
